# Do Systemic Diseases and Medications Influence Dental Implant Osseointegration and Dental Implant Health? An Umbrella Review

**DOI:** 10.3390/dj11060146

**Published:** 2023-06-05

**Authors:** Francesco D’Ambrosio, Alessandra Amato, Andrea Chiacchio, Laura Sisalli, Francesco Giordano

**Affiliations:** 1 Department of Medicine, Surgery and Dentistry, University of Salerno, 84081 Salerno, Italy; 2 Department of Neuroscience, Reproductive Science and Dentistry, University of Naples Federico II, 80138 Naples, Italy

**Keywords:** implant osseointegration, implant success rate, implant survival, implant loss, peri-implantitis

## Abstract

The aim of this umbrella review is to evaluate what are the most common medications and systemic diseases that can affect bone–implant integration, the success rate and survival rate of dental implants, peri-implant tissue health, and implant loss. Systematic reviews, with meta-analysis or not, about how systemic diseases and medications influence dental implant osseointegration, survival rate, success rate, and peri-implant diseases, published only in the English language, are electronically searched across the most important scientific databases. The present umbrella review includes eight systematic reviews, and osteoporosis and diabetes are the most investigated pathologies. Systemic diseases, such as neurologic disorders, HIV, hypothyroidism, cardiovascular diseases, and drugs, such as beta blockers, anti-hypertensives, or diuretics do not show a decreased rate of implant osseointegration. It seems that drugs, such as proton-pump inhibitors (PPIs) or serotonin reuptake inhibitors (SSRIs), negatively affect implant osseointegration. Few studies compare the effects of drugs and systemic diseases on the parameters considered in this overview. It is important to underline how the results of this review need to be validated with subsequent and more reviews.

## 1. Introduction

Implantology is one of the most widespread and predictable treatments to rehabilitate patients with partial or complete edentulism in dentistry [1]. Implant surgery is a real surgical procedure of the jaw bones, which concerns both hard and soft tissues [2,3] and consists of inserting a fixture in the maxillary or mandibular bone in order to integrate the implant with the bone [4,5,6,7].

The clinical success of dental implants depends on osseointegration, which is a biological process consisting of the strict contact of living bone with an endosseous implant [1] and lasts about 3 or 4 months in the mandibular arches and 4 or 6 months in the maxillary arches [4,5].

However, several factors can influence implant placement and the osseointegration process, such as a worsening of bone quantity and quality and problems associated with systemic medical conditions, respectively [8,9,10].

Some international studies have pointed out that the average success rate of implant insertion and osteointegration is over 98%, considering over 1,200,000 implants inserted; this success, however, drops to 85% in smokers [11,12,13].

It is very important to underline how the systemic health of a patient and any pharmacological or radiotherapy therapies may influence the implant treatment [5].

To obtain correct osseointegration of the implants, it is important that there is no interference with the physiological function of the biological activities that must take place during bone healing, where new bone is introduced by osteoblasts and current bone is reabsorbed by the osteoclasts [14].

Anything that can interfere with or alter bone repair and bone healing can cause problems in osseointegration, which can determine peri-implant complications or premature implant loss [15].

Many people have chronic diseases and take systemic drugs, which can influence the success of implant therapy [15].

Among the various drugs that interfere with bone metabolism, the most studied ones are certainly antiresorptive medications, among which are bisphosphonates. These drugs, which have a very long half-life, inhibit the remodeling of the bone and are administered for the treatment of oncological or metabolic bone diseases [16,17].

Bisphosphonates are calcium-binding drugs that decrease the incidence of fractures and increase bone density. Bisphosphonates have often been associated with bone lesions in the jaw, with signs and symptoms even very serious and disabling [18].

However, there are also other systemic conditions and drugs, which interfere with osseointegration and influence implant success and survival rate, implant loss, and peri-implant tissues, that have been described, such as endocrine–metabolic disorders (diabetes and hypothyroidism), neurological diseases, cardiovascular diseases, non-steroidal anti-inflammatory drugs (NSAIDs), glucocorticoids, and many others [19,20,21].

Although data in the literature are scarce and controversial, the aim of this umbrella review is to investigate what are the most commonly used medications that can affect bone–implant integration, success rates, and survival rates of dental implants, peri-implant tissue health, and implant loss.

## 2. Materials and Methods

### 2.1. Study Protocol

This umbrella review was conducted according to the PRISMA (Preferred Reporting Items for Systematic Reviews and Meta-analyses) statement [22] and was registered in the PROSPERO (International Prospective Register of Systematic Reviews) systematic review register (code ID: CRD42023397955), as suggested by Booth et al. [23].

With regard to the research strategies of the question formulation and study selection records, PEO (Population–Exposure–Outcome) questions were considered—a modified version of the PICO model [24,25].

The PEO question is formulated in the following way:

P—Population: people with dental implants taking any type of systemic drug.
E—Exposure: effect of systemic diseases and systemic drugs.
O—Outcomes: implant osseointegration, implant success rate, implant survival, implant loss, peri-implantitis.

### 2.2. Search Strategy

**Figure 1:**
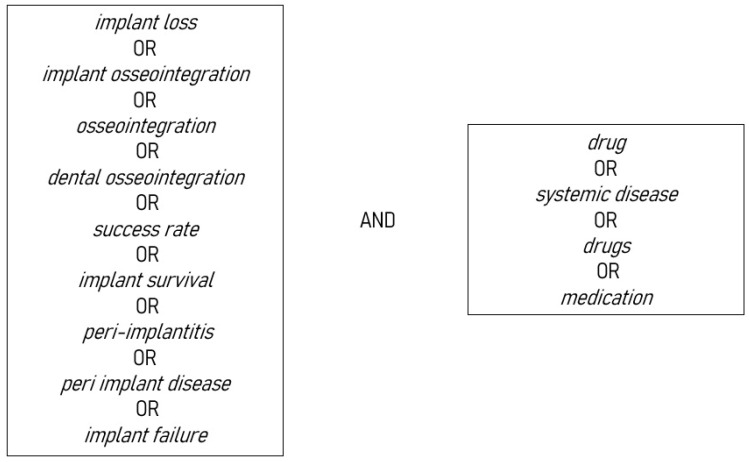
Keywords used for electronic search databases.

All kinds of systematic reviews, with and without meta-analysis, about how systemic diseases and medications influence dental implant osseointegration, survival rate, success rate, and peri-implant diseases were electronically searched until 1 February 2023. The reviews, published only in the English language, were searched in the PROSPERO register, MEDLINE/PubMed, BioMed Central, Scopus, and the Cochrane Library, by two authors (F.D.A.) and (A.C.) using the keyword search settings with Boolean operators shown in Figure 1.

To search the main scientific databases, the filters “Review” on Scopus library and “Systematic Review” and “Meta-analysis” on the MEDLINE/PubMed database were applied. No filters were applied on BioMed Central database, on the Cochrane library, or on the PROSPERO register.

### 2.3. Study Selection and Eligibility Criteria

The main data were extrapolated from the main scientific databases, including articles from the last 10 years, to obtain results only about newer medications.

Inclusion and exclusion criteria, established at the start of the research and listed below, were applied to screen the results.

Inclusion Criteria:

- Systematic review about subjects with dental implants who take any type of systemic drugs.
- Systematic review with or without meta-analysis.
- Articles published in English.
- Reviews published in the last ten years.

Exclusion Criteria:

- Not systematic review with or without meta-analysis.
- Articles not published in English.
- In vitro or animal review.

The duplicates were eliminated thanks to the Zotero reference manager tool, and the titles obtained were evaluated by two authors (A.C.) and (F.D.A).

The two authors independently read the abstracts, and the most relevant ones were chosen in order to obtain the complete text.

A third reviewer (F.G.) was consulted in case of doubts or disagreements.

The bibliographies of the included reviews were also carefully screened for any titles relevant to this umbrella review.

No restrictions were applied to the number and type of studies included in the included systematic reviews.

### 2.4. Data Extraction and Collection

Review data were mainly obtained from two authors (A.A.) and (L.S.), who, however, involved a third reviewer (F.D.A.) in case of perplexity.

From all systematic reviews that were included in this umbrella study, the following data were extrapolated:

- Author, year of publication, reference, name of the journal, and study quality;
- Number and kind of included studies;
- Characteristics of drug intake or diseases assessed;
- Main outcomes;
- Conclusions.

In detail, the outcomes examined in this review were implant osseointegration, implant success rate, implant survival, implant loss, and peri-implantitis.

### 2.5. Data Synthesis

The main features of included reviews, such as author, year of publication, reference, journal of publication, kind of review, study quality, number and design of included studies, the main type of drugs intake or disease evaluated, outcomes, main results, and conclusions, which were considered in this umbrella review, were presented in tabular form and summarized through a narrative synthesis.

Data were synthesized using Microsoft Excel software 2019 (Microsoft Corporation, Redmond, WA, USA).

### 2.6. Assessment of Quality and Risk of Bias

The systematic reviews included in this umbrella review were qualitatively assessed using the Assessing the Methodological Quality of Systematic Reviews (AMSTAR) tool 2, accessible online on 22 November 2022 (https://amstar.ca), which analyzes the quality of systematic reviews of randomized and/or non-randomized studies [26].

## 3. Results

### 3.1. Study Selection

From the first keywords search on the main electronic scientific databases, 5488 records were identified: 137 from BioMed Central, 2538 from MEDLINE/PubMed, 2713 from Scopus, 21 from the Cochrane Library databases, and 79 from the PROSPERO registry.

A total of 1250 duplicates were excluded, and 40 titles were excluded, too, because they were not in the English language, resulting in 4198 abstract titles. Of the 4198 abstract titles, only 18 abstracts were chosen, which were deemed relevant to the present review. Of these 18 abstracts, the full texts were analyzed, and it was decided to exclude 10 articles because there were 3 reviews that were not relevant to this overview and 7 articles that did not meet the inclusion criteria.

**Figure 2:**
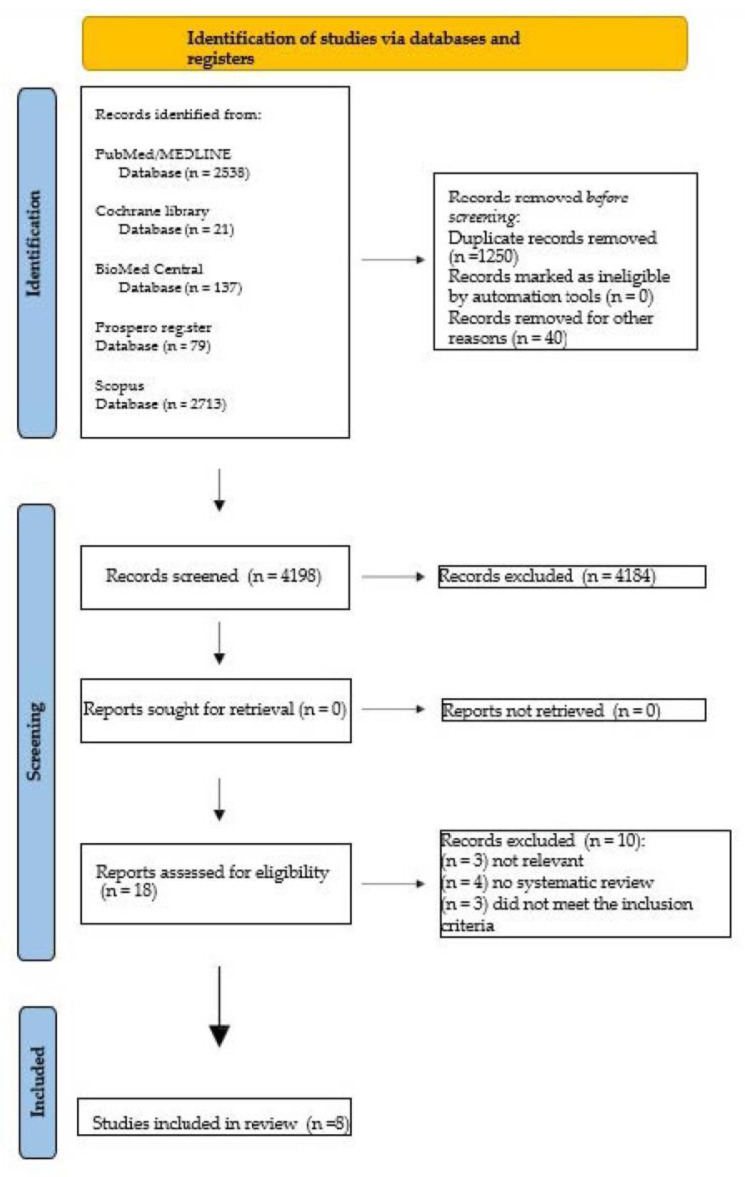
PRISMA flowchart of the screening process.

The PRISMA flowchart of the screening process is illustrated in Figure 2.

The characteristics (author, year, and reason for exclusion) from the excluded studies are synthesized in Table 1.

In the end, a total of 8 systematic reviews were described in this overview.

### 3.2. Studies’ Characteristics and Qualitative Synthesis

The features of the included reviews are summarized in Table 2.

All studies were published in the English language between March 2017 and July 2022.

### 3.3. The Influence of Systemic Drug Intake or General Diseases Evaluated on the Outcomes Considered in This Umbrella Review

The drugs taken or the pathologies considered by the included studies are shown in Table 3, and for each one, the parameters analyzed by the authors were considered (if any).

Many reviews included in this umbrella review considered the effect of bone resorption drugs on outcomes, such as implant osseointegration, implant success rates, implant survival, implant loss, and peri-implantitis.

From the results obtained, it seems that antiresorptive drugs do not significantly influence implant osseointegration, implant success rates, implant survival, and implant loss compared to patients who do not take such drugs.

Stavropulos et al., however, described that patients who underwent hormone replacement therapy (HRT) reported higher implant loss rates compared to controls [42].

No differences were observed among diabetic and non-diabetic patients by Aghaloo in osseointegration rates; instead, Monje described that hyperglycemic subjects, whether smokers or not, were associated with a high risk of peri-implantitis but not with a high risk of peri-implant mucositis [40].

It is more complicated to draw conclusions and analyze the results on other medical conditions or drug intake due to the lack of sufficient data.

Aghaloo et al. described that systemic diseases and medications, such as neurologic disorders, HIV, cardiovascular diseases, anti-hypertensives, diuretics, beta blockers, and hypothyroidism, did not show a decreased rate for implant osseointegration [36].

However, Aghaloo described that drugs, such as SSRIs and PPIs, showed a negative effect on implant osseointegration [36].

**Figure 3:**
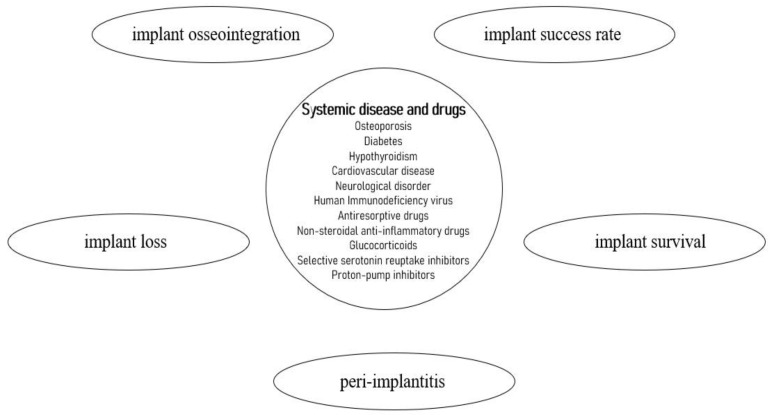
This diagram shows the main pathologies or drugs in relation to the outcomes analyzed in this review.

The correlation between systemic pathologies or systemic drugs and the main outcomes considered in this review is shown in Figure 3.

### 3.4. Quality and Risk of Bias Assessment of Included Systematic Review

Many studies were classified as low or moderate quality, and one was of critically low quality, using the Assessing the Methodological Quality of Systematic Reviews (AMSTAR) 2 tool [26], as illustrated in Table 4.

## 4. Discussion

Dental implants are an efficient and predictable therapeutic alternative for inserting one or more missing teeth [1].

However, systemic conditions and the medications used to treat systemic conditions could affect osseointegration or implant health.

In the literature, there are numerous studies that highlight how some systemic disorders appear to be a contraindication for dental implant osseointegration and for implant health [15,29,32,36,37,44,45]. This overview included reviews that analyzed the influence of only some pathologies and drugs on osseointegration and peri-implant health.

### 4.1. The Main Outcomes of This Umbrella Review (Implant Osseointegration, Implant Success Rate, Implant Survival, Peri-Implantitis, and Implant Loss)

#### 4.1.1. Osseointegration

One of the most important outcomes evaluated in this review was osseointegration, which is defined as the strict contact of bone with an endosseous implant [1], so the interface between the titanium implant and bone host has a very important role. The osseointegration process begins with the deposition of proteins, ions, and other important biological components, such as polysaccharides and proteoglycans, from the titanium oxide layer [10,46,47]. Subsequently, immune system cells, such as macrophages, neutrophils, and osteoprogenitor cells, especially osteoblasts, advance onto the bone–implant interface, and after the initial remodeling, the osteoblasts begin to affix new bone [48,49]. The process of osseointegration takes 3 to 5 months to be adequate enough.

All drugs that possess anabolic properties—such as parathyroid hormone (PTH) peptides, vitamin D, simvastatin, prostaglandin EP4 receptor antagonist, and strontium ranelate—and those with anti-catabolic properties, such as calcitonin, RANK/RANKL/OPG system, bisphosphonates, and selective estrogen receptor modulators (SERMs), could affect this important process [29].

#### 4.1.2. Implant Success Rate, Implant Survival Rate, Implant Loss, and Peri-implantitis

The other outcomes analyzed in this review were the implant success rate, implant survival rate, peri-implantitis, and implant loss.

Dental implant success is not easy to describe; mobility and pain are the primary outcomes to evaluate implant health. Marginal bone loss around implants (MIBL), suppuration, and probing depths are the other criteria to evaluate the success of dental implants [50]. However, the success criterion that is most frequently described is the survival rate of the dental implant, i.e., whether the implant is still present in the oral cavity or has been removed [51]. Peri-implantitis is a disease caused by plaque affecting the tissues around dental implants and is characterized by a progressive inflammation of the peri-implant mucosa with the consequent progressive loss of the supporting bone [12,52]. The incidence of peri-implantitis is one of the most frequent causes of implant failure and consequent implant loss. A large number of therapeutic protocols have been described for the management of peri-implantitis, such as laser or the use of local or systemic antibiotics [53,54,55,56].

### 4.2. The Influence of Most Important Systemic Diseases and Drugs Evaluated on the Outcomes Considered in This Umbrella Review

#### 4.2.1. Metabolic Bone Disease

Metabolic bone diseases, such as osteopenia and osteoporosis, are pathologies particularly frequent in the elderly population. Osteoporosis affects more than 200 million individuals worldwide, mainly postmenopausal women. Osteoporosis is a disease that leads to a progressive loss of bone mass, increasing the risk of fractures and bone fragility. Increased trabecular space could lead to higher implant failure. For this reason, osteoporosis is also considered a risk factor for dental implant therapy [57]. However, not all authors agree that osteoporosis can compromise rehabilitation treatments with dental implants [57], but in the literature, it is well documented that a grade 4 bone density has a greater risk of dental implant failure [58].

Antiresorptive medications are the most used drugs to treat osteoporosis [59]. These drugs down-regulate osteoclast activity, causing a decrease in the bone resorption process. Calcitonin, RANK/RANKL/OPG system, bisphosphonates, and selective estrogen receptor modulators (SERMs) are the most used antiresorptive medications [29]. Bisphosphonates are antiresorptive medications that inhibit osteoclast activity, conserving bone density and strength [60]. Numerous bisphosphonates, such as pamidronate, alendronate, zoledronic acid, ibandronate, and risedronate, have been evaluated concerning the osseointegration of implants [29].

However, these drugs have been shown to have side effects; the most frequent is osteonecrosis of the jaws (ONJs), characterized by progressive bone destruction and necrosis of the mandibular and/or maxillary bone tissue. In fact, numerous studies have described how subjects to whom these drugs had been administered presented ONJ in the absence of a radiant treatment [61].

Jaw bone infections or bone trauma caused by the insertion of a dental implant increases the risk of ONJ in patients taking bisphosphonates [62].

Dental implantology could represent a very efficient choice that considerably improves the quality of life, both for subjects not receiving antiresorptive drug therapy and for patients undergoing antiresorptive therapy. However, it is very important to underline the importance of informing patients on anti-absorptive drug therapy contraindications, such as the possibility of developing medication-related osteonecrosis of the jaw (MRONJ), which has a huge negative impact on the quality of life of these patients [63,64].

However, the risk of developing ONJ in patients who have taken antiresorptive drugs and who undergo oral surgical procedures, such as the placement of dental implants, is not known, as pointed out by the American Association of Oral and Maxillofacial Surgeons [65].

From the results of this review, it emerged that most of the reviews included did not observe statistically significant differences, considering the osseointegration process, between patients treated with antiresorptive drugs and subjects not treated.

In agreement with the results obtained by Aghaloo et al. [36], there are also the results obtained by Chadha et al. and by Javed et al. [18,45].

Najeeb et al. demonstrated that the presence of bisphosphonates on the surface coating can have a positive effect on the osseointegration of dental implants [66].

Antiresorptive drugs do not seem to affect the survival and success of dental implants. The reviews included in our analysis showed no difference between patients taking antiresorptive drugs and those who are not.

Aghaloo’s review showed a 98% implant survival rate, which is comparable to patients without osteoporosis [36]. This result is confirmed by a recent systematic review by de Medeiros et al., where 10 studies analyzed in this meta-analysis showed a failure rate of 4.70% on 702 implants entered in patients with osteoporosis, which is similar to the 3.57% failure rate on 4114 entered in healthy subjects. Thus, they concluded that there are no significant differences in implant failure rates between implants in patients with systemic osteoporosis and implants in patients without osteoporosis [67].

Papadakis et al., in a recent systematic review, analyzed the success rate and safety of dental implantology in patients who took antiresorptive drugs but could not provide an adequate conclusion [41]. This result is due to the fact that controversial studies were analyzed in their review; in fact, Zahid et al. and Kasai et al. concluded that oral bisphosphonates could reduce dental implant osseointegration and, thus, increase their failure rate, but the other studies showed a very low failure rate for dental implants, with no statistically significant differences between the rates of patients treated with bisphosphonates and the control groups [68,69,70].

The bisphosphonate intake studies included in a meta-analysis by Stavropoulos et al. showed no significant differences regarding implant loss between the patient taking bisphosphonates and the patient not taking bisphosphonates; moreover, the implant success rate varied for cases from 85.7% to 100%, which was superimposable to 84.6% of 100% controls [42].

Controversial results on implant loss were obtained from two studies; in the first study, Yip et al. (2012) reported an odds ratio (OR) of 2.7 for bisphosphonate intake in test subjects compared to controls [71], while a report by Al-Sabbagh and Robinson et al. (2015) described that controls had a higher risk of implant loss than cases, with an OR of 9.2 for controls [72]. The authors also assessed other factors, such as graft failure, marginal bone loss, the development of MRONJ, and the presence of peri-implantitis [71,72].

In general, studies that considered peri-implant marginal bone loss or level did not show relevant differences between cases and controls. This meta-analysis concluded that low-dose oral bisphosphonates used in the treatment of osteoporosis present no contraindications for implant therapy. Patients treated with bisphosphonates are not at an increased risk of losing implants or experiencing complications and/or implant failures compared to patients who have dental implants and who do not take bisphosphonates [42,73].

There are also other drugs for the treatment of osteoporosis. In particular, for patients with osteoporosis who are unresponsive to or intolerant of other drugs, an anabolic agent, 1–34 PTH or teriparatide [74], which has been shown to increase osseointegration of implants, may be used.

Tao et al. (2015) showed that 1–34 PTH, given together with simvastatin, a drug used for the control of fats in the bloodstream and which seems to improve osseointegration, has a greater positive influence on osseointegration than the use of drugs alone [75,76]. Javed et al., in a recent review, concluded that intermittent PTH therapy promotes new bone formation around implants [29,77].

#### 4.2.2. Endocrine–Metabolic Disorder

Diabetes mellitus is one of the most widespread and most common endocrine–metabolic disorders in the world. It is a multifactorial metabolic imbalance distinguished by chronic high glycemia caused by changes in the secretion of the hormone insulin with alterations in carbohydrate, protein, and lipid metabolisms [78,79]. About 150 million people worldwide had this pathology in the 1980s, and in 2008, the number of sick subjects increased significantly, reaching 350 million [78]. The association between diabetes and periodontal disease and the consequent tooth loss, delayed wound healing, and reduced response to infections is well demonstrated in the literature [80].

Moreover, in the World Workshop 2017 on Peri-implants, Schwarz et al. concluded that studies in the literature are controversial in stating that diabetes is a risk factor for peri-implantitis [81]. Ferreira et al. reported an odds ratio (OR) of 1.9; Tawell et al. reported that subjects who had a mean HbA1c level ≤ 7% did not show implants with peri-implantitis, but in patients with high HbA1c levels (7% to 9%), 6 out of 141 implants showed peri-implant disease [82,83].

In contrast, Costa et al. did not find any difference between diabetic patients and healthy patients with regard to the presence of peri-implant disease, as also confirmed by other the authors of [84,85,86,87].

Regarding the correlation between diabetes and osseointegration, a review conducted by Aghaloo et al. [36] showed that are no differences between healthy subjects and diabetic patients; the same results were also obtained by Javed et al. and Naujokat et al. [80,88]. However, Aghaloo points out that chronic states lead to an increase in cytokines and other pro-inflammatory mediators, causing a reduction in the coupling between osteoblasts and osteoclasts [36]. Aghaloo’s review, therefore, presents an important limitation, as she considers osseointegration at the time of definitive loading, at least 3 months after the surgical implant procedure [36].

In any case, Aghaloo points out that long-term studies show an increase in MIBL and peri-implantitis in diabetic patients with dental implants [36].

The studies included in this review do not show statistically significant differences between diabetic and non-diabetic patients with regard to osseointegration; however, the results are described in the short term and not in the long term, a factor which could, instead, change the results obtained [36].

Another major metabolic disorder is hypothyroidism, a condition in which the thyroid gland is unable to synthesize enough hormones to meet the needs of the entire body. Thyroid hormone maintains adult bone mass and affects bone metabolism by stimulating the production of insulin-like growth factor 1, which increases osteoblast formation [36,89,90].

Aghaloo describes that for bone metabolism, hypothyroidism has been associated with an increased risk of fracture, with delayed bone regeneration and fracture repair [36]. However, Aghaloo describes that studies do not demonstrate statistically significant differences in the rate of implant failures between hypothyroid subjects and control patients [36].

#### 4.2.3. Non-Steroidal Anti-Inflammatory Drugs (NSAIDs)

Non-steroidal anti-inflammatory drugs (NSAIDs) are drugs that have anti-inflammatory and analgesic action. They can be taken for short periods, as in the case of dental care, or for long periods, as in autoimmune diseases [91].

NSAIDs act by indirectly inhibiting the production of prostaglandins, which play a key role in the inflammatory cascade. These drugs, in fact, act as selective inhibitors of COX-2 and do not allow arachidonic acid to be converted into prostaglandins [15]. Inflammation, which is enhanced by the effect of prostaglandins, determines that there is a greater supply of cells responsible for bone formation [29]. NSAIDs, by blocking the production of prostaglandins, indirectly determine poor differentiation of osteoblasts at the expense of greater bone apposition [32]. The importance of COX-2 was demonstrated by Chikazu et al. (2007) [92], who, in a study performed on mice, demonstrated how rodents that were deficient in COX-2 had poor bone formation around the implants compared to mice that did not present this deficiency [29].

In another randomized controlled study in humans, it was found that oral administration of ibuprofen at a dosage of 600 mg 4 times a day for a period of 7 days did not cause any bone loss after 3–6 months of use [93].

A 2014 retrospective study [94] found that more failures in implant osseointegration occurred in patients who took NSAIDs after surgery.

Furthermore, more peri-implant bone loss and more implant failures were found in patients treated with NSAIDs [71].

However, in the literature, there are discordant points of view, but in general, it would be preferable to avoid taking NSAIDs before or after implant placement for the management of post-operative pain and edema [15].

From the reviews included in this overview, it emerged that there is not sufficient scientific evidence to be able to state whether their use can affect the osseointegration of dental implants [39,43]. It should, however, be noted that the results of studies where NSAIDs were taken for long periods should not be compared to NSAID use for short periods, such as postoperative surgical management.

Furthermore, the doses of NSAIDs, which often differ from one study to another, should be considered, leading to difficulties in discussing the results [32]. Finally, it is important to underline that there are substantial metabolic differences between humans and animals, which can explain the different results obtained by the various authors [32].

#### 4.2.4. Glucocorticoids

Glucocorticoids are a class of drugs used to reduce inflammation in chronic diseases, such as autoimmune diseases, asthma, chronic bowel disease, and rheumatoid arthritis. However, from what emerges in numerous studies, even these drugs, in the case of prolonged treatments with high doses, have side effects, including reduced bone formation [32].

In fact, glucocorticoids seem to negatively influence bone remodeling since they act on osteoblasts, the cells responsible for bone formation, causing apoptosis, and promote the differentiation of adipocytes from bone marrow cells [32].

Prolonged use of these drugs can, therefore, also negatively affect the optimal osseointegration of dental implants [95,96].

One of the main absolute contraindications to the prolonged use of glucocorticoids is the placement of dental implants in the oral cavity [97,98].

There are, however, conflicting studies on the effects of glucocorticoids on osseointegration and implant healing. The osseointegration of dental implants associated with glucocorticoid intake in humans has not been investigated in long-term, high-quality clinical studies, for which more randomized controlled trials are expected.

#### 4.2.5. Antidepressant Medications: Selective Serotonin Reuptake Inhibitors (SSRIs)

Depression, a globally prevalent mental illness, is associated with significant disability and reduced quality of life. As a cause of depression, low levels of serotonin have been implicated, so selective serotonin reuptake inhibitors (SSRIs) have been used successfully to treat this disease [15,99,100]. With regard to the former, some clinical studies have shown a relationship between them and a reduction in bone mineral density and a greater risk of bone fracture [101,102]. This is because osteoblasts and osteoclasts express 5-Hydroxytryptamine (5-HT) receptors and can be exposed to autocrine, paracrine, and endocrine 5-HT pathways [36].

Indeed, the reviews included in our analysis showed a higher rate of dental implant failure in patients taking SSRIs. A review by Chappuis et al. showed a difference in failure rate of 7.5% between SSRI and non-SSRI users [37], while a review by Aghaloo et al. showed a dental implant survival rate ranging from 89.4% to 94.4% in patients suffering from SSRIs versus patients undergoing no medication with a rate ranging from 95.4% to 98.15 % [36]. These results also confirm a review by Cheng et al., who showed a difference in implant failure rate of 7.5% [44].

A recent meta-analysis found that SSRI use is associated with a significantly increased risk of fractures [103]. It has been suggested that serotonin receptors present in osteocytes, osteoblasts, and osteoclasts may be activated by SSRIs and, thus, alter their function [104]. Taking all these factors into consideration, Wu et al. hypothesized that SSRIs may have a negative effect on the osseointegration of dental implants [105]. From a retrospective cohort study of 916 implants in 490 patients, it was found that patients with SSRIs had an increased risk of failure for osseointegrated implants [15].

#### 4.2.6. Proton-Pump Inhibitors (PPIs)

Proton-pump inhibitors (PPIs) are a class of drugs that act on the production of gastric acid, resulting in a reduction. Some authors, however, have described how PPIs have been linked to an increased risk of bone fractures, perhaps by altering calcium absorption [36].

Prolonged use of PPIs, leading to a reduction in acidity, may adversely affect the absorption of vitamin B12, magnesium, iron, and, especially, calcium [36].

The reviews included in our analysis showed a higher rate of dental implant failure in patients taking PPIs. A review by Chappuis et al. [37] showed that the implant failure rate among patients taking PPIs was different from those not taking PPIs by 4.5%, while a review by Aghaloo et al. [36] showed a dental implant failure rate between 12% and 6.8% in patients taking PPIs compared to patients without a dressing, at a rate between 4.5% and 3.2%. These results also confirm a review by Cheng et al. [44], who showed a difference in the implant failure rate of 4.5%. Vinnakota et al. and Chawla et al., in their reviews, showed that there was a signal between PPI and dental implant failure, as shown in our analysis [106,107].

#### 4.2.7. Cardiovascular Disease

Cardiovascular diseases, increasingly widespread in industrialized countries, directly influence bone healing and osseointegration through the supply of oxygen to peripheral tissues, the activity of cells of the immune system, such as macrophages, and the decrease in fibroblast activity [108].

The studies included in a review by Aghaloo et al. showed that cardiac disease and hypertension did not adversely affect early implant failures [36].

Furthermore, it appears that antihypertensive drugs, such as beta blockers or ACE inhibitors, may even positively influence the implant failure rate, as shown in reviews by Chappuis and Aghaloo [36,37].

Wu also demonstrated a lower implant failure rate in patients taking antihypertensive drugs, showing a failure rate of 0.6% in patients taking antihypertensive drugs compared with 4.1% in patients not taking such drugs [108].

#### 4.2.8. Neurological Disorder

Neurological diseases, which afflict the elderly population, usually negatively affect survival and implant success, as these patients present with poor oral hygiene, oral parafunctions such as bruxism, unhealthy habits, and behavioral problems [36].

The development of recent technologies and their use in medicine have allowed the use of dental implants even in patients suffering from neurological diseases, but there is little evidence to support this.

The reviews included in our analysis showed that there is no direct evidence that the rate of osseointegration is reduced in patients with neurological disorders [36].

#### 4.2.9. Human Immunodeficiency Virus (HIV)

The HIV virus causes acquired immune deficiency syndrome (AIDS), which is an infectious disease caused by the HIV virus; it attacks and weakens the immune system.

HIV-positive individuals have impaired bone metabolism caused by impaired calcium/vitamin D absorption, lower testosterone levels, and highly active antiretroviral therapy (HAART) [109].

Oliveira showed that patients infected with HIV and on HAART do not have success and survival rates for dental implants that are very different from those who do not have this pathology or are on HAART [36,110].

A recent systematic review, conducted by Ata-Ali et al. pointed out that the failure of dental implants was comparable between HIV-positive patients and healthy patients [111].

### 4.3. Limitations of this Study and Distorted Quality of the Research

The heterogeneity found in the reviews included in this overview, which report different pathologies that can influence the health of implants and osseointegration, does not allow for conducting statistics on the data obtained. Furthermore, most of the studies were rated as low or moderate quality, and one was critically low quality, by the Methodological Quality Assessment Tool of Systematic Reviews (AMSTAR) 2 [26]. The few systematic reviews on the subject have focused on different drugs and pathologies, except for antiresorptive drugs, which have been analyzed by as many as four authors. The skewed quality of eight included reviews and the lack of adequacy of the RCTs included in the reviews analyzed pose other limitations to this umbrella review.

It should also be noted that the included reviews have different settings and are at a moderate to high risk of bias.

It should be considered that the various studies included in the reviews analyzed in this overview did not always highlight any bias in their studies. For example, the association of pathologies or systemic drugs with smoking or alcohol must be kept in mind and represents a bias in the results. This is because cigarette smoking and most modern smoking devices, such as electronic cigarettes or heat-not-burn tobacco, could be a cofactor for peri-implant pathologies [12,13,112].

In fact, the 2017 World Workshop on periodontal and peri-implant diseases drafted a new classification that highlighted smoking as a risk factor for periodontal disease and peri-implantitis [81].

The reviews included include only some systemic conditions and some medications; however, in a general overview, we need to keep in mind that there are also other systemic conditions and other medications that could negatively affect implant success and osseointegration, including chemotherapeutic agents or immunosuppressive drugs such as cyclosporine A, or even positively, such as statins used to control blood cholesterol levels [15].

## 5. Conclusions

Eight systematic reviews were included in this umbrella review. Few studies compare the effects of drugs and systemic diseases on the parameters considered in this overview: implant osseointegration, implant success and survival rate, peri-implantitis, and implant loss.

From this overview, no direct evidence emerges that the intake of antiresorptive drugs can influence the osseointegration of dental implants. However, keep in mind what the possible side effects might be, such as in the case of osteonecrosis of the jaws (ONJs).

Conflicting results on diabetes emerge from this overview, which, however, need to be carefully evaluated and contextualized, as most of the studies included in the reviews are short-term studies and include patients with well-controlled diseases, while long-term studies and patients with uncontrolled diabetic disease have shown worse results for diabetes, as also attested by the recent 2017 World Workshop on peri-implants, where diabetes was included among the systemic risk factors for periodontal disease and peri-implantitis.

One of the main absolute contraindications to the placement of dental implants in the oral cavity is the prolonged use of glucocorticoids, due to their negative influence on bone remodeling.

Particular attention should also be paid to users of selective serotonin reuptake inhibitors (SSRIs) and proton-pump inhibitors (PPIs), which, from recent but still not sufficient studies, seem to negatively affect osseointegration and increase the risk of bone fractures.

Regarding cardiovascular disease, neurological disorders, hypothyroidism, HIV, or the use of drugs, such as non-steroidal anti-inflammatory drugs (NSAIDs), there is no clear evidence, and the study results are conflicting. Further studies are needed to evaluate the long-term effects on implant osseointegration, implant success, survival rate, and implant loss.

However, an important consideration is to frame the patient systematically before each implant surgery maneuver in order to evaluate the risks and benefits of the implant surgery itself.

Furthermore, always be updated and consult the most recent guidelines on new drugs and on what the implications on the insertion or on implant success may be.

Molecular and genetic research on this front is also very important to find an explanation on drugs affecting bone formation and further develop better agents with neutral or positive effects on implant osseointegration.

Further studies, including long-term ones, are needed to compare how systemic pathologies and/or systemic drugs can influence implant osseointegration, implant success, survival rate, and implant loss.

## Figures and Tables

**Table 1:** Excluded studies and motivation.

Authors, Year	Motivation
Apostu, 2018 [27]	No systematic review
De Oliveira, 2020 [28]	No systematic review
Apostu, 2017 [29]	No systematic review
Pokrowiecki, 2018 [30]	Not relevant
Thirunavukarasu, 2015 [31]	Not relevant
Ouanounou, 2016 [15]	Did not meet the inclusion criteria
Fu, 2012 [32]	No systematic review
Basudan, 2018 [33]	No human studies
Clementini, 2013 [34]	Not relevant
Kellesarian, 2017 [35]	Did not meet the inclusion criteria

The table shows authors, year, and motivation for exclusion.

**Table 2:** Main features of included reviews: author, year of publication, reference, journal of publication, kind of review with meta-analysis (if present). Number and design of included studies; type of drug intake or disease evaluated; main outcomes and conclusions.

Author, Year Reference Journal Meta-Analysis	Number and Design of Included Studies	Type of Drug Intake or Diseases Evaluated	Outcomes	Conclusions
Aghaloo, 2019 [36]; *Int J Oral Maxillofacial Implants*; systematic review	Osteoporosis, not taking BPs: 2 PSs, 2 CCSs Osteoporosis, taking BPs: 10 RSs, 3 CCSs, 4 PSs Diabetes: 5 RSs, 5 CCSs, 10 PSs	Osteoporosis, diabetes, cardiovascular disease or hypertension, Parkinson’s disease or neurocognitive disease, rheumatoid arthritis, hypothyroidism, HIV, depression, anti-hypertensives or diuretics, PPIs, and SSRIs	Implant osseointegration	It seems that patients with osteoporosis, diabetes, hypothyroidism, HIV, neurocognitive disease, rheumatoid arthritis, and cardiovascular disease do not have a decreasing rate of implant osseointegration. Instead, SSRIs and PPIs show a negative effect on osseointegration.
Chappuis, 2018 [37]; *Clinical Oral Implants Research*; systematic review and meta-analysis	NSAIDs: 5 RCTs, 2 RSs SSRI: 2 RSs PPI: 2 RSs BP: 5 RSs, 1 CCS, 1 PS; AHTN: 1 RS	BP, NSAIDs, SSRIs, PPIs, AHTNs	Implant failure, MIBL	The authors show that PPIs and SSRIs negatively influence implant success and oral BPs did not yield significance upon implant failure.
Fiorillo, 2022 [38]; *BMC Oral Health*; systematic review	5 RCTs, 3 MSs, 1 CT	BPs	BoP, PPD, MIBL, mobility of dental implant, ISQ, BMD, implant survival, TE, soft tissue condition	The authors underline that there are no statistically significant differences in MIBL, despite the fact that implants associated with BPs show better results. The authors underline pharmacological prophylaxis before implant insertion.
Luo, 2018 [39]; *International Journal of Implant Dentistry*; systematic review	2 in vitro studies, 3 CTs, 8 animal studies	NSAIDs	Implant osseointegration	NSAIDs do not negatively influence osseointegration in human studies, although these results contrast with in vitro and in vivo animal studies.
Monje, 2017 [40]; *Journal of Clinical Periodontology*; systematic review and meta-analysis	5 PSs, 1 RSs, 6 CSSs	Hyperglycemia	Peri-implant mucositis, peri-implantitis	The risk of peri-implantitis is higher in hyperglycemic subjects than normoglycemic subjects but with not statistically significance differences.
Papadakis, 2023 [41]; *J Oral Implantology*; systematic review	4 PSs, 7 CCSs 21 RSs	ARDs	Success rate, survival rates	This review describes that ARDs do not influence the success and survival rates of dental implants.
Stavropoulos, 2018 [42]; *Clinical Oral Implants Research*; systematic review and meta-analysis	BP intake: 8 CSs, 10 cohort studies, 6 CCSs HRT intake: 5 CSs, 2 CCSs MRONJ associated with implants: 7 CSs	ARDs, BPs, HRT	Implant loss, failure of grafting procedure, MIBL, MRONJ, peri-implantitis	This review showed that low-dose oral BPs do not compromise implants and do not show complications/ failures as compared to patients with no BP intake. High-dose BPs or other ARDs show a high risk for MRONJ, but few studies are about implant therapy.
Werny, 2022 [43] *International Journal of* *Implant Dentistry*; systematic review	13 animal studies, 3 CTs, 2 human RSs	Vitamin D	Implant osseointegration	Vitamin D deficiency negatively influences osseointegration and its supplementation improves osseointegration in animals. Limited information is available for human implant osseointegration.

Abbreviations: MRONJI—medication-related osteonecrosis of the jaw; MIBL—bone implant marginal loss; NSAIDs—non-steroidal anti-inflammatory drugs; SSRIs—serotonin reuptake inhibitors; PPIs—proton-pump inhibitors; ARDs—antiresorptive drugs; AHTNs—anti-hypertensives; BPs—bisphosphonates; HIV—human immunodeficiency virus; HRT—hormone replacement therapy; RCT—randomized clinical trial; PSs—prospective studies; RSs—retrospective studies; CCSs—case-control studies; CSSs—cross-sectional studies; MSs—multicentric studies; CT—clinical trial; CSs—cohort studies; PPD—periodontal probing depth; BoP—bleeding on probing; ISQ—implant stability quotient; BMD—bone mineral density; TE—thread exposure.

**Table 3:** Characteristics of outcomes, drug intake, and general diseases and the main results from included studies.

Outcomes	Author, Year	Drug Intake and General Diseases	Main Result
Osseointegration	Aghaloo (2019) [36]	ARDs	No differences in patients with or without osteoporosis with antiresorptive therapy.
		Diabetes	No differences were seen among diabetic and healthy patients.
	Luo et al. (2018) [39]	NSAIDs	Could not be adequately estimated.
	Werny et al. (2022) [43]	Vitamin D	There was slight evidence that vitamin D supplementation improves implant osseointegration in humans.
Implant survival rate	Aghaloo et al. (2019) [36]	Osteoporosis	The pooled estimated was 98% (with a confidence interval of 95%).
		Neurologic disorder	The implant survival rate was 86%.
	Papadakis et al. (2023) [41]	ARDs	Antiresorptive medication did not reduce the success rate of dental implants or implant survival rates.
	Chappuis et al. (2018) [37]	Anti-hypertensive, diuretics, or beta blockers	The analysis could not be performed.
Implant loss rate	Stavropoulos et al. (2018) [42]	ARDs	No differences in patients with or without osteoporosis with antiresorptive therapy.
		HRT	HRT intake studies reported a higher implant loss rate (9.1–27.3%) compared to controls (7.4–16.1%).
Implant success rate	Papadakis et al. (2023) [41]	ARDs	ARDs did not reduce the success rate of dental implants or implant survival rates.
Implant failure rate	Chappuis et al. (2018) [37]	ARDs	No differences in patients with or without osteoporosis with antiresorptive therapy.
		Anti-hypertensive, diuretics, or beta blockers	The analysis could not be performed.
		SSRIs	It seems that the test (SSRI intake) group had a significantly higher risk than the control group.
		PPIs	Implant failure rates were higher in the test group compared to the control group (*p* < 0.01).
	Aghaloo et al. (2019) [36]	HIV	A 0.8% failure rate compared to 100% failure in non-HIV patients.
		Cardiovascular disease	No statistically significant differences were found among test and control groups.
		Hypothyroidism	No differences were found among healthy and diseased patients.
		Anti-hypertensive, diuretics, or beta blockers	In patients who took medication, implant survival rate was 99.4% versus 95.9% in patients not taking these medications.
		SSRIs	A lower implant survival rate of 89.4% to 94.4% vs. 95.4% to 98.15% in patients not taking these drugs was shown.
		PPIs	PPI users had an increased implant failure rate of 12% to 6.8% vs. 4.5% to 3.2% in PPI non-users.
Bone marginal loss	Fiorillo et al. (2022) [38]	ARDs	No differences in patients with or without osteoporosis with antiresorptive therapy.
Peri-implant mucositis or peri-implantitis	Monje et al. (2017) [40]	Diabetes	The risk of peri-implantitis in hyperglycemic subjects was statistically significantly higher than normoglycemic subjects.

Abbreviations: NSAIDs—non-steroidal anti-inflammatory drugs; SSRIs—serotonin reuptake inhibitors; PPIs—proton-pump inhibitors; ARDs—antiresorptive drugs; HIV—human immunodeficiency virus; HRT—hormone replacement therapy.

**Table 4:** Level of evidence of systematic reviews with meta-analysis included according to the AMSTAR 2 tool.

Level	Description	Aghaloo, 2019 [36]	Chappuis, 2018 [37]	Fiorillo, 2022 [38]	Luo, 2018 [39]	Monje, 2017 [40]	Papadakis, 2023 [41]	Stavropoulos, 2018 [42]	Werny, 2022 [43]
High	No or one non-critical weakness: the systematic review provides an accurate and comprehensive summary of the results of the available studies that address the question of interest.								
Moderate	More than one non-critical weakness: The systematic review has more than one weakness but no critical flaws. It may provide an accurate summary of the results of the available studies that were included in the review.		✓					✓	
Low	One critical flaw with or without non-critical weaknesses: the review has a critical flaw and may not provide an accurate and comprehensive summary of the available studies that address the question of interest.	✓		✓	✓		✓		✓
Critically low	More than one critical flaw with or without non-critical weaknesses: the review has more than one critical flaw and should not be relied on to provide an accurate and comprehensive summary of the available studies.					✓			

## Data Availability

Not applicable.
